# Evaluation of Functionality of Warning System in Smart Protective Clothing for Firefighters

**DOI:** 10.3390/s21051767

**Published:** 2021-03-04

**Authors:** Anna Dąbrowska, Grażyna Bartkowiak, Rafał Kotas

**Affiliations:** 1 Department of Personal Protective Equipment, Central Institute for Labour Protection—National Research Institute, 90-133 Lodz, Poland; grbar@ciop.lodz.pl; 2 Department of Microelectronics and Computer Science, Lodz University of Technology, 90-924 Lodz, Poland; rafal.kotas@p.lodz.pl

**Keywords:** smart clothing, smart PPE, wearables, warning systems, firefighters

## Abstract

Recent achievements in the field of miniaturization of electronics have led to a significant interest in its application into the protective clothing for firefighters in order to improve their safety and health. However, up to now there are not any requirements and standardized test methods enabling confirmation of safety of use and functionality of these systems in work environment. In the paper, an importance of evaluation of such smart wearable system in predicted utility conditions is highlighted. Three variants of the specially developed personal warning systems (PWSs) for integration with either health status or environmental sensors to be used in smart protective clothing for firefighters are presented, i.e., with LCD display, LED diodes and vibrating element. For the purpose of evaluation a new testing methodology was developed. The PWSs were evaluated on a basis of perception of warning signals by firefighters in simulated utility conditions including temperature, light, noise, fog and performed activities. In the case of marching, even 75% of signals generated by PWS with LCD display were not received. Physical activity did not influence on functionality of PWS with vibrating element. For the PWS with LED diodes, the signal was received statistically significantly quicker than in the case of other PWS and the mean value of voice response time was below 3 s.

## 1. Introduction

The profession of a firefighter is often associated with work in extremely difficult conditions, subject to a number of hazards that can affect their health and safety. These include thermal factors, fire smoke, the possibility of explosions, flammable and toxic substances, noise, darkness and poor visibility (and associated location issues), work at height, as well as physical hazards associated with trips, falls and crushing [1,2,3]. The work of a firefighter is also associated with a large physical effort and burden associated with, inter alia, the mass of personal protective equipment used. Serious threats can also result from stress, adverse weather conditions, as well as high variability in the work environment. This all affects the psychophysical state of firefighters [3].

Smith et al. [4] found that firefighters are exposed during the service to exhausting physical activity, emotional stress and environmental pollution that can affect the cardiovascular system and, as a consequence, lead to cardiac arrest. In urban and industrial areas, firefighters are most often called for fire rescue operations. These types of actions can last from several minutes to even several days [5] and are carried out in severe conditions: in the presence of fire, flammable or toxic materials, in potentially explosive atmosphere, in buildings that may collapse. Firefighters work in high temperature, dark, smoky and noisy (due to fire) environment [6]. Due to the speed of fire spreading, sudden, difficult to predict events, e.g., damage to the structure of the object or its components, may occur, as a result of which the firefighter is exposed to injuries, including: fractures, crushes, cut or stab wounds. Even more dynamic are the damages inside the building resulting from the explosion, the shock wave of which also poses a serious threat to firefighters (shards, displacements caused by the blast) [7].

Taking into account such extremely hazardous work environment, stress that accompanies firefighters cannot be omitted. Its level may be also evaluated on a basis of measurements of physiological parameters [8]. Among the physiological parameters, special attention was paid to the variability of the heart rate as a marker of stress in the human body [8,9,10,11]. Both mental and physical activity increases the heart rate. Stress analysis is primarily based on the heart rate variability (HRV). A decrease in HRV for a short period of time is interpreted as an indicator of acute stress. It is also observed that a decrease in HRV is often accompanied by an increase in respiratory rate [12]. Other solution for assessment of stress and fatigue of firefighters during their activities is based on heart rate and respiratory cycle and then use several accelerometers to take related measures [13]. Another physiological parameter that is often used in the analysis of mental stress is the galvanic skin response (GSR), the variability of which is also closely related to the stress stimulus [14]. GSR reflects the change in the electrical properties of the skin under the influence of experienced emotions or a spontaneous response to a given stimulus. The registered increase in skin resistance is a consequence of sweat secretion and the filling of sweat channels that act as resistors. However, it should be noted that in order to obtain more reliable information, a multimodal approach is beneficial, assuming an analysis based on various physiological parameters and their combinations [8,9].

Considering the above data, related to the firefighter’s working conditions and psychophysical burden, it can be considered that monitoring and then signaling hazards in the firefighter’s work environment seems to be almost as important as monitoring his vital signs to ensure safety and health during work. Monitoring systems can be integrated with protective clothing, which is then called smart protective clothing for firefighters, or can be separate modules compatible with it.

Many works related to wearable sensors for firefighters use directly the IoT (Internet of Things) concept [15,16]. Some articles presents systems that are similar to the one presented in this work [17,18]. However, it has to be stated that main focus in those publications is set to the system description without presenting any relevant tests.

An important element of threat or vital functions monitoring systems is signaling that the set threshold values of monitored parameters are exceeded. Signaling should be unambiguous, legible, but at the same time not causing too much absorption of the firefighter and not disturbing his activities. Due to the hazards to which the firefighter is exposed, primarily flame and heat radiation, signaling systems should not increase the risk in this respect.

In the context of smart systems integrated with protective clothing, a lot of research has already been carried out, in particular in the field of solutions for firefighters [19,20,21,22,23,24]. The analysis of the state of knowledge in this area was presented in detail by Dąbrowska [25]. It should be emphasized, however, that so far no requirements and test methods have been developed that in a uniform manner on the European market would allow confirming the safety and functionality of using such smart systems under anticipated conditions of use. Electronic solutions cannot pose an additional threat to firefighters, so the essence of reliability of these solutions is emphasized. In the case of firefighting clothing, in particular the possible impact of thermal factors (i.e., flame, thermal radiation, convective heat) on the proper functioning of electronic systems as well as other environmental conditions during firefighting (i.e., smoke, darkness, noise) should be taken into account. Communication and location systems used in firefighting operations face similar problems. High temperature, noise, dense smoke, gusts of air, obstacles and falling debris impede the spread of radio, ultrasonic and laser signals, which in turn leads to interruption of communication and, as a result, limits the control over the course of action. Therefore, within this manuscript, a new testing methodology of evaluation of smart wearable personal warning systems for firefighters in simulated utility conditions is presented and the results of analysis of the influence of kind of physical activity performed by firefighters on the perception of three kinds of warning signals are shown.

## 2. Materials and Methods

### 2.1. Personal Warning Systems for a Use with Smart Protective Clothing for Firefighters

Currently, communication systems, including information on hazards in the fire service are based on radio communication, which is guaranteed by specially dedicated radios operating in the VHF band. Two-way radios are part of the firefighter’s equipment during operations, and can also be mounted in a fire truck. The most important features that must be met by firefighting radios are reliability, durability and resistance to weather conditions. Supplement to radio communication may be personal warning systems (PWSs), which are intended most often to be mounted on the firefighter’s personal equipment, including they can be combined with personal protective equipment.

In this field, the needs and expectations of firefighters were defined in terms of the appropriate way of signaling hazards and data on the conditions for the potential use of PWS in smart protective clothing was collected [26]. The most important requirements that firefighters have pointed out and which must be taken into account when designing signaling systems include the following:

- Signaling modules should be light and implemented in a way that does not adversely affect the ergonomics of protective clothing;
- They should not absorb excessive attention during operation, at the same time their signal should be recognizable;
- The way the signaling modules work should involve the use of hands as little as possible.

Given the preferences of firefighters, in a cooperation with the Department of Microelectronics and Information Technology of the Lodz University of Technology and ILED sp. z o.o., three variants of personal warning systems were developed:

- PWS with LCD display;
- PWS with LEDs;
- PWS with vibration element.

The design of the developed PWSs is shown in Table 1. The signaling modules of individual PWS variants were placed in specially designed textile covers, in the form of bands, made of flame retardant and heat resistant materials. Proper functioning of the developed PWS in conditions of increased ambient temperature to 70 °C was confirmed by 5-min exposure tests in the laboratory drying machine. Temperature and time of exposure was selected according to the requirements for radio communicators for fire fighters.

### 2.2. Testing Methodology

#### 2.2.1. Tested Object

Developed three variants of personal warning systems were subjected to utility tests. The tests were carried out using a developed computer application, enabling the control of signal parameters generated by individual variants of the PWS. Characteristics of signals generated during the tests were selected on the basis of surveys carried out among firefighters. In the case of PWS with LCD display, two hazard messages were presented: CH—chemical (on a yellow background), T—temperature (on a red background). In the case of PWS with LEDs, hazard signaling was carried out by flashing red LEDs (luminous intensity 550–700 mcd) with maximum brightness (level 255), the signal period was 200 ms, while the low state was 100 ms. In the case of PWS with a vibration motor—the signaling was progressing—falling (triangular), low level had minimal intensity (level 0), high level had maximum intensity (level 225), rise time was 1000 ms and fall time 1000 ms.

**Figure 1:**
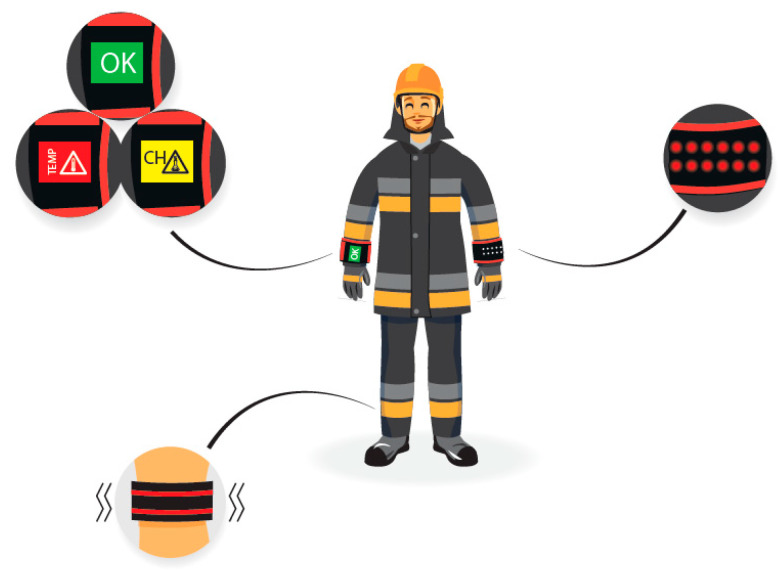
A scheme of location of PWS during the utility tests.

Utility tests were carried out with the participation of 8 firefighters equipped with two-piece protective clothing, helmet, gloves and footwear with three variants of the PWS—with LCD display and LED diodes (on the forearms) and with a vibration motor on the leg (Figure 1).

#### 2.2.2. Testing Procedure

In order to evaluate functionality of the particular PWSs in predicted utility conditions, a new special test methodology was designed. Utility tests included performing selected five physical activities that simulated the real activities performed during firefighting operations in a tenement house (Table 2). The tests were carried out in laboratory conditions at an ambient temperature of 25 °C, relative humidity of 50%, at an air speed of 0.24 m/s. This test procedure was developed as a result of consultations with firefighters.

After entering the laboratory, participants were instructed regarding the course of the experiment, and then signed a voluntary consent form to participate in the study. Then, firefighters put on protective clothing along with footwear and a helmet, after which three variants of the PWS were put on in the following locations: PWS with LCD display on the right forearm on top of the clothing, PWS with LEDs on the left forearm on the top of clothing and PWS with a vibrating element on the right leg above the ankle under clothing.

Utility tests were carried out at individual stations according to the schedule (Table 3), where: LCD-T—temperature hazard message from PWS with LCD display, LCD-CH—chemical hazard message from PWS with LCD display, LED—hazard signal from PWS with LEDs and Vibration—hazard signal from PWS with vibrating element. In addition, during tests at station 1, an explosion sound was played through the sound system in the laboratory room. Ensuring the assumed environmental conditions (i.e., light temperature and intensity, and smoke intensity) was possible thanks to the specially designed software that allows synchronized control of environmental conditions from a computer level (see Section 2.2.3).

During the experiment, in the laboratory room, there were only a study participant and a person supervising the study and controlling its duration at individual stations. The rest of personnel involved in the study was in the external control room with a voice communication and view on the laboratory room.

During the experiment, hazard signals were generated until confirmation of receipt was received or until 20 s had elapsed. Participants confirmed the reception of the signal using a wireless voice communication system. After performing utility tests at individual stations, participants assessed the functionality of individual PWS variants in the survey.

#### 2.2.3. Apparatus and Tools Used

**Figure 2:**
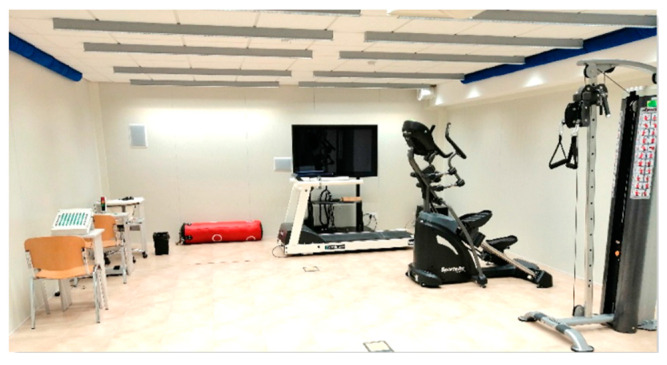
A view on the laboratory room.

Utility tests were carried out in the Research and Demonstration Laboratory at the Department of Personal Protective Equipment of CIOP-PIB, enabling the simulation of various conditions of the expected use of new kinds of personal protective equipment solutions. The laboratory room in which the tests were carried out is a thermally insulated room (Figure 2), providing the ability to control climate effects in terms of temperature, relative humidity and air velocity, as well as lighting (by adjusting the intensity and temperature of light) and sound system (through the built-in spatial sound system). In addition, the laboratory is equipped with a BeamZ S1500 smoke generator, enabling smoking the room with a substance safe for human health. The essence of the Laboratory is also the software that allows synchronized control of environmental conditions from a computer level, located in the control room with a view on the laboratory room.

To simulate the activities carried out during firefighting activities, the following equipment of the Research and Demonstration Laboratory were used: Zebris Rehawalk h/p/cosmos mercury 150/50 type FDM-THM-M-3i treadmill, the SportsArt Fitness S775 (LED) climbing trainer and the TuffStuff Apollo 7000 Series AP-71HL-1/2 upper and lower lift.

For recording the voice response time, the EJEAS VNETPHONE wireless communication system and the DELTA E 100 electronic stopwatch were used. In addition, for each PWS variant the time associated with signal transmission and recording was determined.

The obtained results of the voice response time were subjected to statistical analysis in order to determine the statistical significance of differences between individual PWS variants and stations—conditions of use. Before starting the analysis, the normality of variable distribution was checked using the Shapiro–Wilk test. Due to the failure to meet the normality criterion for distribution, a nonparametric Wilcoxon character sequence test for dependent samples was used for statistical analysis. The level of significance of tests 0.05 was adopted. The statistical analysis was carried out using the STATISTICA 13.1 PL program.

The survey questionnaire consisted of three sections of questions—for each variant of PWS separately in which the influence of particular physical activities on functionality of PWS was evaluated (single choice questions: easy perception of signal, difficult perception of signal, no influence). In the last question, research participants rated the functionality of three PWS variants in general using a scale from 1 to 10, where 1 is the worst and 10 is the best score.

## 3. Results and Discussion

### 3.1. Test Results of the Voice Response Time to the Signal

The results of the average voice response time as well as the time of signal transmission from the moment of its generation to the moment of its perception for individual PWS are summarized in the tables below (Table 4 and Table 5, respectively).

Based on the obtained test results (Table 4), it can be stated that the shortest average signal response time was obtained in the case of PWS with LEDs at station 3 (upper lift)—1.63 s, and the longest—in the case of PWS with LCD display on station 1 (treadmill) for both the message with information about temperature and chemical hazards (12 s). It should be noted, however, that the waiting time for response to the signal was up to 20 s and in the case of PWS with LCD display during exercise at station 1 as much as 75% of messages in the case of temperature hazard and 62.5%—in the case of chemical hazard, were not received at all.

Comparing the average voice response times at individual stations, it can be stated that in the case of the first station (treadmill walking) the highest values were obtained for all PWS variants. Therefore, it can be concluded that the perception of signals during this type of physical activity is difficult, because in the case of PWS with LCD display the difference between the signal transmission time (Table 5) and the response time (Table 4) was over 9 s, which is over 3 more than alone message transfer time. Comparing the response time results for individual PWS variants at station 1, no statistically significant differences were found.

In the case of PWS with LCD display, the shortest response times were obtained for the temperature hazard message at station 4 (transition to all fours)—5.33 s, while for the chemical hazard message at station 2 (climbing trainer)—5.75 s. It should be noted that the second longest response time in the case of PWS with LCD display was obtained in the case of a chemical hazard message at station 4 (8.60 s), while in the case of a temperature hazard message—at station 2 (8.25 s). These results suggest that messages from the PWS with LCD display are difficult to receive regardless of the physical activity performed and the time of voice response to the threat signal was almost random.

The shortest average voice response time regardless of the performed physical activity was achieved in the case of PWS with LEDs. The lowest value (less than 2 s) was obtained in the case of station 4 (moving on all fours). This result is justified because during the exercise the hands were arranged in the field of view, therefore the generated signal (flashing red light) was easy to see. The statistical analysis showed that the difference in response time to the signal generated by the PWS with LEDs at station 1 and station 4 is statistically significant.

Comparing the received voice response times at individual stations, it can be stated that, with the exception of station 1, the signal generated by PWS with LEDs was statistically significantly faster received than in the case of other PWS.

### 3.2. Survey Results

**Figure 3:**
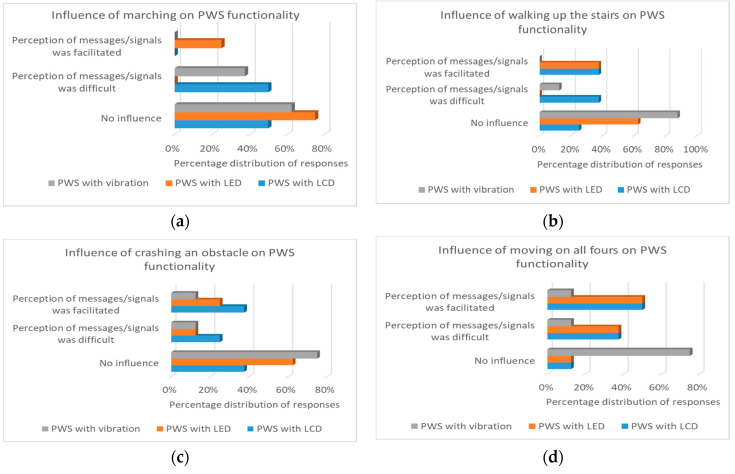

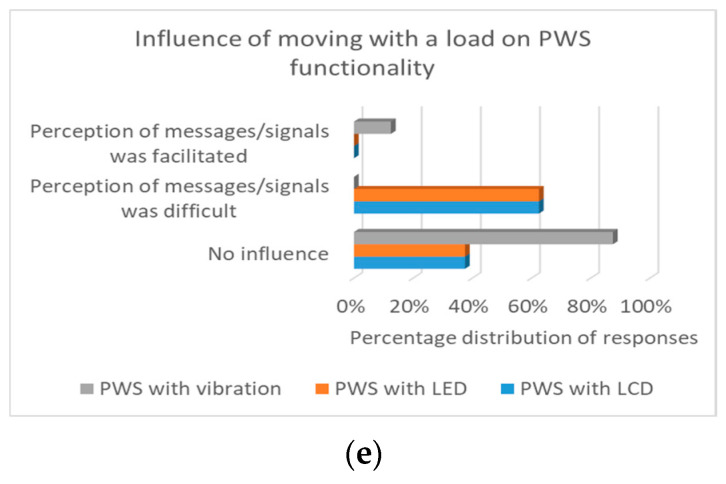
Survey results of the influence of the type of physical activity on the functionality of the PWS: (**a**) treadmill; (**b**) climbing trainer; (**c**) upper lift; (**d**) moving on all fours; (**e**) moving with a load.

**Figure 4:**
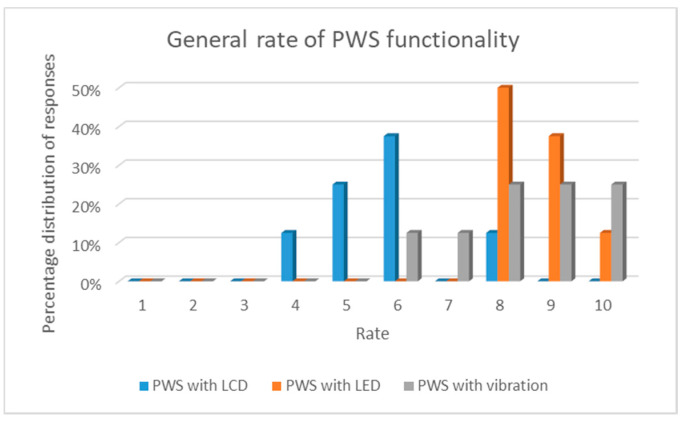
Survey results of the overall PWS functionality assessment, where 1—the lowest rating, 10—the highest rating.

The results of the surveys carried out are presented in the form of charts with percentage distribution of responses. In Figure 3a–e are presented particular results of the first survey question about the influence of particular physical activities on PWS functionality. While in Figure 4—general rate of PWS functionality.

The conducted research on the impact of the type of physical activity on the functionality of PWS (Figure 3a–e) indicates that only in the case of PWS with a vibrating element, regardless of the performed physical activity, most of the respondents stated that there was no effect of the given activity.

In the case of PWS with LCD display, as an exercise that hindered the receipt of messages, the respondents indicated walking on the treadmill (50.0%) and moving with a load (62.5%), while moving on all fours was indicated as activity facilitating the reception of the message (50.0%).

In the case of PWS with LEDs, respondents indicated walking on the treadmill (75.0%), exercise on the climbing trainer (62.5%) and on the upper lift (62.5%) as activities that did not affect the functionality of the PWS, while moving on all fours was indicated as activity facilitating signal reception (50.0%), and switching with load—as activity hindering signal reception (62.5%).

Analyzing the results of the overall PWS functionality assessment (Figure 4), it can be stated that the highest score was given to PWS with a vibration element (25.0%—rating 10). In the case of this system, 25% of respondents each gave a rating of 8, 9 and 10. In the case of PWS with LEDs, slightly less respondents gave a rating of 10 (12.5%), but more—8 (50.0%) and 9 (37.5%). As a consequence, the system with LEDs received the highest average rating of PWS functionality—8.625 ± 0.696 (average ± standard deviation). In the case of PWS with a vibrating element, this rating was slightly lower and amounted to 8.375 ± 1.317. PWS with LCD display received a much lower average overall rating—5.710 ±1.161. For this system variant, most of the ratings awarded were at level 6 and lower.

Interviews with firefighters after the end of the study indicate that the participants positively assess the proposed PWS; however, the preferences regarding the choice of signaling type are divided. Although the PWS with LCD display received the lowest rating, firefighters reported that this solution is good because it allows to convey more complex messages. However, the problem is the way the message is displayed, because hazard signaling should not require focusing on the PWS and the possibility of the message appearing. A hazard signal should be perceptible regardless of environmental conditions and physical activities performed. In this respect, firefighters rated PWS with a vibrating element best. Advantageous in this case is also the possibility of attaching both on the wrist and on the leg above the ankle, because the preferences of firefighters in this respect vary. Some appreciated the fact that attaching the PWS to the leg ensures that there is no need to focus attention on the hands and there is no additional equipment that can be exposed to various types of mechanical damage. The second group, in turn, preferred PWS mounted on hands, because of the ease of putting on, e.g., on the way to the action in a fire engine. Therefore, it is not possible to make a clear judgment about which setting is best. Despite the fact that firefighters rated PWS with LEDs the highest, interviews show that in practice they prefer to combine PWS with LCD and LEDs rather than PWS with LEDs alone. This solution would provide a combination of easy message reception thanks to LEDs and the possibility of transmitting a more complex message thanks to the LCD display.

## 4. Conclusions

Three PWS variants were tested in the Research and Demonstration Laboratory with the participation of eight firefighters. During the tests, firefighters dressed in protective clothing, a helmet, gloves and footwear, and the developed PWS performed physical activities (five exercise stations) corresponding to the selected conditions of use. These activities were carried out under strictly defined environmental conditions, i.e., temperature, relative humidity, air velocity, lighting (determined by temperature and intensity), smoke and noise. During the exercise, hazard signals for individual PWS variants were generated at each station from a computer application. In order to assess the functionality of the developed PWS time of voice response to the signal were recorded during the tests. In addition, at the end of the experiment the participants completed a questionnaire.

Based on the tests, a statistically significant impact of the type of physical activity on the PWS functionality was found, especially in the case of PWS by visual methods. In the case of walking on a treadmill, as much as 75% of temperature hazard messages were not received by study participants. Research has shown, however, that signals generated from PWS with LEDs are statistically significantly faster received by users compared to PWS with LCD display and PWS with vibrating element (p-value at the level of 0.028). It should be emphasized, however, that in the case of PWS with a vibration element, no impact of the conditions of use (in terms of given physical activity) on its functionality was found. As a result of the research, it was found, however, that the proposed PWS solution with LCD display is not sufficient in its current form to be used during firefighting operations, because the messages transmitted by it were not noticed during the tests in simulated conditions of use. However, interviews with firefighters show that this solution is well evaluated, as it allows the transmission of more precise messages than the other two methods. However, it should be used together with additional signaling (e.g., by means of diodes or a vibrating element) that would draw attention to the display.

Proposed methodology proved that a proper construction of wearable system is not enough for guaranteeing its proper functioning in real utility conditions and this aspect is of particular importance in the case of protective applications. The most accurate sensors will not fulfill their assumed function when the information about the danger will not be received by the user. Therefore, utility tests that include simulated utility conditions and participation of potential end users are significant added value to the safety-oriented research projects that can verify both the ergonomics of the designed new solutions, as well as indicate any shortcomings in their functioning that require improvements before implementation to the market. Moreover, a direction of implementation of objective methods in utility tests that may also include biofeedback methods should be also promoted to limit the discrepancies between the subjective opinion of the test participant and the reality.

## Figures and Tables

**Table 1:** Personal warning systems (PWSs).

Description of PWS	A View of PWS
Personal warning system with LCD display: PWS consists of a board with a 2.4 “LCD display, which also serves as a programmable processor (microcontroller), Wi-Fi communication module and LCD display. This system is powered by a USB connector using a powerbank. As a result of consultations with firefighters, the PWS allows the transmission of three messages: no danger (“OK”), temperature alarm, chemical alarm. As specified in the technical documentation provided by its manufacturer the LCD module can operate in the ambient temperature range −10 to +60 °C (with possible periodic exposures to maximum temperature rating of −20 to +70 °C).	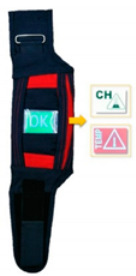
Personal warning system with LED diodes: This system consists of two flexible lines of RGB LEDs (6 pcs in each line), a microcontroller and a LiPo battery. It allows to adjust the light color (RGB model), brightness and frequency of light pulses transmitted by the diodes by specifying the expected high state time (diode on) and low state time (diode off). As specified in the technical documentation provided by its manufacturer the WS2812 diode junction can operate in the ambient temperature range −25 to +80 °C	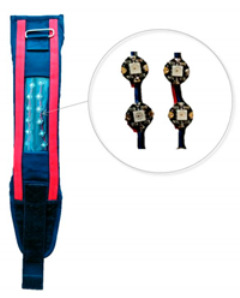
Personal warning system with vibrating element: PWS is built of a system of 5 vibration micro motors connected in parallel, a microcontroller and a LiPo accumulator. It provides the ability to adjust in terms of vibration (triangular or rectangular) and the intensity of vibration as a function of time. As specified in the technical documentation provided by its manufacturer the vibration micro motors can operate in the ambient temperature range −10 to +60 °C	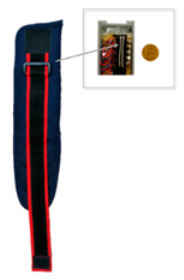

**Table 2:** Description of the stations with the characteristics of the physical activities performed.

No	Station	Characteristics of Physical Activity	Photo
1	Getting to the place of incident	Treadmill walking at 5 km/h for 10 min	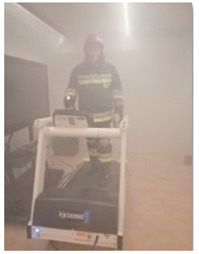
2	Walking up the stairs	Exercise on a climbing trainer at a speed of approx. 40 str/min, stride length 30–50 cm, time 3 min	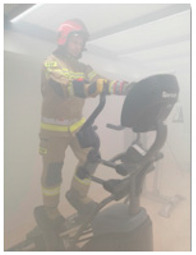
3	Crashing an obstacle	Exercise on the upper lift with a load 18 kg, 3 sets of 10 repetitions for 3 min	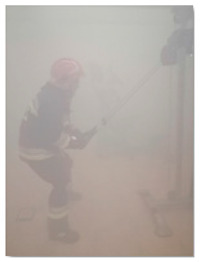
4	Reaching the injured	Moving on all fours, a total distance of 15 m in 3 series of 5 m for 3 min	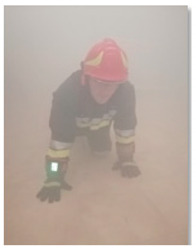
5	Transport of injured	Moving with a load 21.5 kg, a total distance of 15 m, in 3 series of 5 m for 3 min	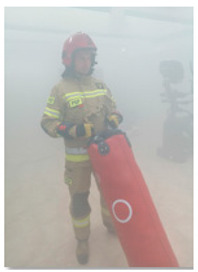

**Table 3:** Schedule of the experiment at individual stations.

Signal	Time, min:s	Light Intensity, lux	Light Temperature, K	Smoke Generator Efficiency, %
LCD—T	1:15	1450 (to 5. min)	6500 (to 5. min)	15 (to 3. min)
Noise 1	2:00			
Vibration	3:30			
LED	4:20			
LCD—CH	6:30	10 (from 5. min)	2700 (from 5. min)	0 (from 3. min)
LED	7:20			
Vibration	8:50			
Noise 2	9:30			
LCD—T	0:30	10	2700	0
LED	1:15			
Vibration	2:00			
LCD—CH	2:45			
LED	0:30	10	2700	0
LCD—CH	1:15			15 (from 1. min)
LCD—T	2:00			0
Vibration	2:45			
LCD—CH	0:30	10	2700	0
Vibration	1:15			
LED	2:00			
LCD—T	2:45			
LED	0:30	10	2700	0
LCD—CH	1:15			
Vibration	2:00			
LCD—T	2:45			

**Table 4:** Test results of the voice response time to the signal.

Station	Signal	Voice Response Time, s	Missed Signals, %
Station 1	LCD-T	12.00 ± 4.24	75.0
	Vibration	7.17 ± 3.87	0.0
	LED	3.33 ± 1.86	0.0
	LCD-CH	12.00 ± 9.64	62.5
	LED	3.38 ± 1.30	0.0
	Vibration	6.75 ± 4.77	0.0
Station 2	LCD-T	8.25 ± 4.57	50.0
	LED	2.50 ± 0.93	0.0
	Vibration	6.13 ± 2.95	0.0
	LCD-CH	5.75 ± 2.87	50.0
Station 3	LED	2.50 ± 1.20	0.0
	LCD-CH	7.71 ± 3.40	12.5
	LCD-T	6.71 ± 7.30	12.5
	Vibration	5.13 ± 3.40	0.0
Station 4	LCD-CH	8.60 ± 1.67	37.5
	Vibration	5.63 ± 5.01	0.0
	LED	1.63 ± 0.74	0.0
	LCD-T	5.33 ± 2.80	25.0
Station 5	LED	2.13 ± 0.83	0.0
	LCD-CH	6.67 ± 2.25	12.5
	Vibration	5.50 ± 4.57	0.0
	LCD-T	6.14 ± 2.04	12.5
Mean values from all stations	LCD-T	7.69 ± 2.64	35.0
	LCD-CH	8.15 ± 2.41	35.0
	LED	2.58 ± 0.68	0.0
	Vibration	5.83 ± 0.63	0.0

**Table 5:** Results of transmission time (TT) for particular PWS.

TT, s	LCD-T	LCD-CH	LED	Vibration
Mean value	2.58	2.54	<1 s	1.06
SD	0.15	0.07	-	0.12

## Data Availability

The data presented in this study are available on request from the corresponding author.
